# Investigation of Three-Dimensional Structure and Pigment Surrounding Environment of a TiO_2_ Containing Waterborne Paint

**DOI:** 10.3390/ma12030464

**Published:** 2019-02-02

**Authors:** Fei Yang, Bo Chen, Teruo Hashimoto, Yongming Zhang, George Thompson, Ian Robinson

**Affiliations:** 1 School of Materials Science and Engineering, Tongji University, Shanghai 201804, China; 1610413@tongji.edu.cn (F.Y.); zym126@tongji.edu.cn (Y.Z.); i.robinson@ucl.ac.uk (I.R.); 2 London Centre for Nanotechnology, University College London, London WC1H 0AH, UK; 3 Key Laboratory of Performance Evolution and Control for Engineering Structures of the Ministry of Education, Tongji University, Shanghai 200092, China; 4 School of Materials, The University of Manchester, Manchester M13 9PL, UK; t.hashimoto@manchester.ac.uk (T.H.); george.thompson@manchester.ac.uk (G.T.); 5 Division of Condensed Matter Physics and Materials Science, Brookhaven National Laboratory, Upton, NY 11973, USA

**Keywords:** three-dimensional (3D) structure, TiO_2_ pigment, voids, waterborne paint, serial block-face scanning electron microscopy (SBFSEM)

## Abstract

Serial block-face scanning electron microscopy (SBFSEM) has been used to investigate the three-dimensional (3D) structure of a cured waterborne paint containing TiO_2_ pigment particles, and the surrounding environment of the TiO_2_ pigment particles in the cured paint film was also discussed. The 3D spatial distribution of the particles in the paint film and their degree of dispersion were clearly revealed. More than 55% of the measured TiO_2_ particles have volumes between 1.0 × 10^6^ nm^3^ and 1.0 × 10^7^ nm^3^. From the obtained 3D images, we proposed that there are three different types of voids in the measured cured waterborne paint film: voids that exist in the cured paint themselves, voids produced by particle shedding, and voids produced by quasi-liquid phase evaporation during measurement. Among these, the latter two types of voids are artefacts caused during SBFSEM measurement which provide evidence to support that the pigment particles in the cured paint/coating films are surrounding by quasi-liquid environment rather than dry solid environment. The error caused by particle shedding to the statistical calculation of the TiO_2_ particles was corrected in our analysis. The resulting 3D structure of the paint, especially the different voids are important for further systematic research, and are critical for understanding the real environment of the pigment particles in the cured paint films.

## 1. Introduction

Due to their good functionality as both a protective or decorative layer and their low production cost, organic paints/coatings are now an essential part of our daily life [1]. They can not only protect structures and components against aggressive external environment including sunlight, water, various chemical and physical attacks, but also provide color and gloss to the substrates [2,3]. Currently, the use of solvent-borne organic paints is widely blamed or even banned in more and more cases since they contain numerous volatile organic compounds (VOCs) and toxic chemicals, making them harmful to the environment and human beings [4,5]. However, waterborne paints use water as solvent, which is not only cheap, but also non-toxic to human beings and the environment, making this type of paint one of the ideal choices for paint manufacturing in the modern coating market [6]; for these reasons, there have been many studies carried out on waterborne paints [7,8].

Owing to having high refractive index, good whiteness and dispersion, non-toxicity, stable physical and chemical properties etc., titanium dioxide (TiO_2_) particles have attracted a lot of research interest and have been utilized in numerous commercial applications including many kinds of paints [9]. Thanks to their compatibility with aqueous dispersions, they are widely used in waterborne paints as well [10]. Due to the excellent chemical–physical stability and mechanical resistance of acrylic resins, a lot of attention has been paid to them as well by different researchers and manufacturers [1,11]. The selected TiO_2_ containing waterborne paint samples were prepared from the AkzoNobel Dulux Trade Weathershield commercial product line with nominal composition of 38% (nominal solid in weight by pigment/(pigment + binder)) TiO_2_ pigment and an acrylic emulsion binder in water solvent, which is also one of the most representative materials in the field of paint research [12]. In the reported study, serial block-face scanning electron microscopy (SBFSEM) [13,14,15] used in the investigation of biological [16,17,18] and material [19,20,21] specimens, was used to reveal the three-dimensional (3D) spatial structure of the TiO_2_ pigment containing waterborne paint. SBFSEM system obtains 3D serially parallel electron micrographs of the TiO_2_ pigmented waterborne paint by imaging the freshly exposed surfaces of the samples that are generated by serial-sectioning using an indoor ultra-microtome. The sectioning cycles were conducted by the ultramicrotome system, with a diamond knife, which is installed in the vacuum chamber of the scanning electron microscope.

The investigated target waterborne paint has good whiteness, chemical inertness, and high refractive index. These properties are related to the 3D structure of the cured paint film, especially the spatial distribution of the TiO_2_ pigment particles and the internal pores. In addition, the costs of the TiO_2_ pigmented waterborne paint is mainly determined by the consumption of TiO_2_ pigment, which is much more expensive than the acrylic resin and solvent, i.e. water, in the paint. A proper distribution of the TiO_2_ particles will reduce the amount of TiO_2_ used, and hence reduce the costs of the paint manufacturing. The investigation of the 3D spatial structure of the cured paint film including the distribution of TiO_2_ pigment particles and voids within the matrix material of paints is reported in this paper. The findings also support that the surrounding environment of the pigment particles in the cured paint films are probably quasi-liquid phase rather than dry solid status based on the analysis of the data. The research provides a powerful approach for studying waterborne paints, and the results are meaningful for the performance optimization and manufacture of the paints.

## 2. Materials and Methods

The measured specimen of TiO_2_ pigmented waterborne paint was first applied on a plastic sheet by brushing. It was then cured at room temperature for one week. The obtained dry paint film was then stored in a paper envelope for another year before being measured by SBFSEM (Thermo Fisher Scientific, Waltham, MA, USA and Gatan Inc., Abingdon, UK).

Before the waterborne paint film is measured by SBFSEM, the sample must be fixed firmly. Sample fixing was done by directly bonding the dry paint film, which was peeled off from the substrate, to a flat aluminum slide using cyanoacrylate glue. After the cyanoacrylate glue was cured at room temperature for a couple of hours, the waterborne paint film with its supporting aluminum slide were trimmed into a pyramid shape using a microtome by a glass knife. The upper surface of the sample was trimmed to approximately 500 µm × 500 µm in dimensions. The resulting pyramid shape sample was then mounted in a Gatan 3View sample holder for 3D imaging measurement, which worked with a FEI field emission gun environmental SEM (FEI QUANTA 250, Thermo Fisher Scientific, Waltham, MA, USA). This SBFSEM measurement was done at 3 kV with 0.524 Torr (70 Pa) chamber pressure. The diamond knife moved along the X axis which is parallel to the upper surface of the sample or the XY plane in the image. A stack of backscattered electron (BSE) micrographs of the waterborne paint sample with a pixel size of 13 nm × 13 nm was obtained after about 1.5 h of continuous measurement. In total, a sample with thickness of 1.5 µm (100 slices) was imaged with a field of view of 13.3 µm × 13.3 µm (1024 × 1024 pixels). 

## 3. Results and Discussions 

**Figure 1:**
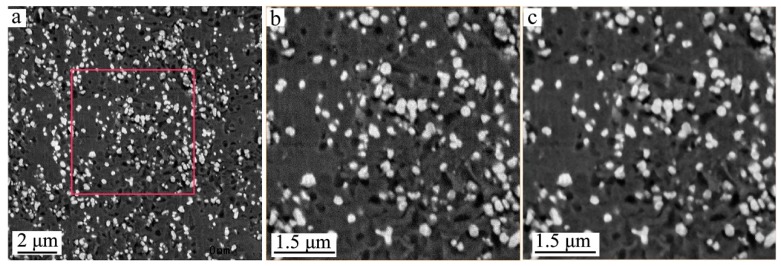
Two-dimensional (2D) backscattered electron (BSE) micrographs of the TiO_2_ pigmented waterborne paint film obtained from serial block-face scanning electron microscopy (SBFSEM) measurement: (**a**) An original BSE micrograph slice of the sample acquired by SBFSEM; (**b**) The zoomed-in image of the part enclosed by the red square, 500 × 500 pixels (6.5 µm × 6.5 µm) large, in panel a; (**c**) The noise reduction processed image of the selective part as shown in panel b. The grey parts are acrylic resin, the white parts are TiO_2_ particles, and the black parts are voids.

Figure 1a presents the first two-dimensional (2D) BSE micrograph of the waterborne paint sample out of the stack of 100 obtained slices by SBFSEM. The part enclosed by the red square in Figure 1a was selected for 3D reconstruction and analysis, which is displayed in Figure 1b. Figure 1c shows the improved image of Figure 1b after noise reduction, which is the data used for 3D image segmentation, rendering, and analysis. In accordance to the principle that the brightness of the BSE micrographs scale with the atomic masses of different composites, in the TiO_2_ pigmented paint sample, the white particles in the BSE micrographs are identified as TiO_2_ particles, all the grey regions within the images are identified as acrylic resin, and the remaining dark parts in the images are identified as voids. At the first glance, the distribution of TiO_2_ particles, acrylic resin, and voids within the sample can be clearly seen in these images. The first step to analyzing the 3D image was to align the BSE micrograph slices of the sample, in order to solve the problem of image shifts caused by the possible sample movements and image drifts. Then the threshold segmentation and label analysis were used to perform 3D structural image analysis of the sample. All the data processing above was done via Avizo which is a 3D image processing software package from Thermo Fisher Scientific, Waltham, MA, USA.

**Figure 2:**
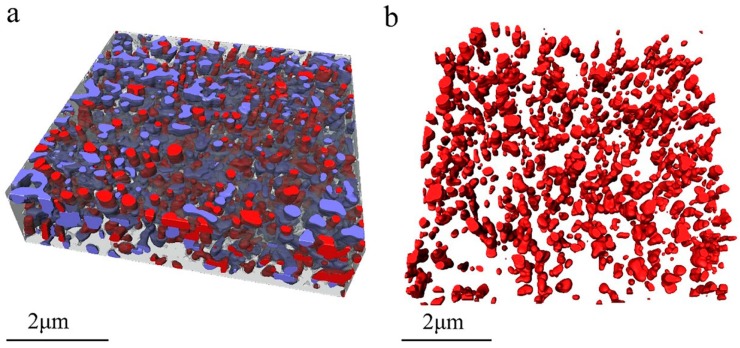
3D rendering images of the sample: (**a**) 3D rendering of the acquired paint volume containing TiO_2_ particles; (**b**) 3D spatial distribution of the TiO_2_ particles only. The translucent grey parts are acrylic resin, the red parts are TiO_2_ particles, and the blue parts are voids.

Figure 2a shows the 3D image of the measured dry waterborne paint film. In Figure 2a and the subsequent figures, the translucent grey parts are acrylic resin, the red parts are TiO_2_ particles, and the blue parts are voids. From these images, the TiO_2_ particles and the voids within the acrylic resin are clearly illustrated in three dimensions. Figure 2b presents the spatial distribution of the TiO_2_ particles only. Although the shape of the TiO_2_ particles (see Figure 2b) look like irregular in three dimensions, the distribution of the TiO_2_ particles are relatively homogenous in the cured paint film.

**Figure 3:**
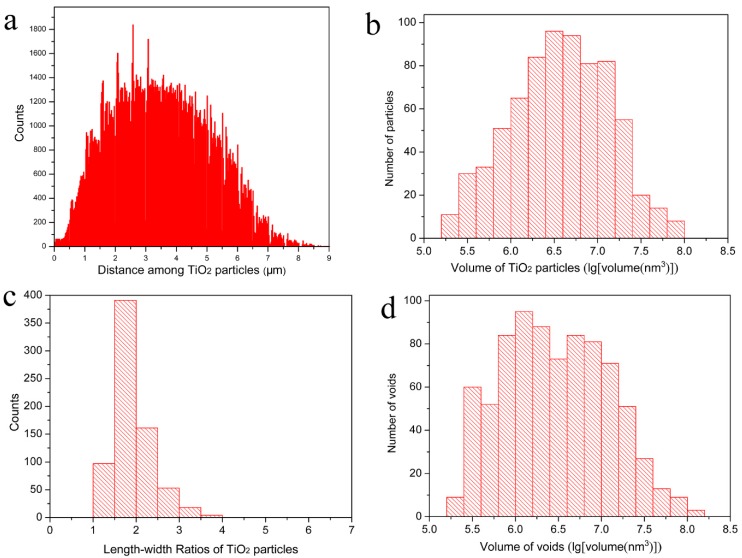
3D characterization of the TiO_2_ particles and voids within the measured waterborne paint film: (**a**) A histogram of distribution of distances among individual TiO_2_ particles; (**b**) A histogram of the volume distribution of the TiO_2_ particles; (**c**) A histogram of the length–width ratios of the measured TiO_2_ particles; (**d**) A histogram of the volume distribution of the voids.

The dispersion of TiO_2_ particles in the cured paint film directly affects the performance of the waterborne paint and the consumption of the TiO_2_ pigment during paint manufacturing. The distribution of the distances among different TiO_2_ particles are clearly presented in Figure 3a; it can be known that the minimum distance between the particles is 124 nm and about 79% of the distances are between 1.5 µm and 5.5 µm with a nearly Gaussian distribution. This means that the TiO_2_ particles have a relatively homogeneous distribution in the cured paint film as shown in Figure 1 and Figure 2 as well. The volumes of the single TiO_2_ particles range from 2.0 × 10^5^ nm^3^ to 8.7 × 10^7^ nm^3^ (see Figure 3b); they are also listed in Supplementary Table S1 numerically. These TiO_2_ particles have a relatively narrow size distribution, and for about 58% of them, the volume is between 1.0 × 10^6^ nm^3^ and 1.0 × 10^7^ nm^3^. The length–width ratios of the TiO_2_ particles are presented in Figure 3c, which shows that the length–width ratios values of all the particles are between 1.3 and 3.9, and 67% of them are between 1.3 and 2.0. This indicates that most of the TiO_2_ particles are rice-shaped, and they have a rather unique size range as well which is in a good line with the information obtained from the supplier. Here, the length–width ratio of a particle is the ratio of its maximum Feret diameter against its minimum Feret diameter [22]. The volume of the voids in the measured dry waterborne paint film are presented in Figure 3d which shows that the volumes of all the voids are between 1.8 × 10^5^ nm^3^ and 1.3 × 10^8^ nm^3^, and 74% of them are between 1.0 × 10^6^ nm^3^ and 1.0 × 10^8^ nm^3^. By analyzing the statistical figures in Supplementary Table S1, it can be obtained that the average volume, length, and length–width ratios of the TiO_2_ particles are 8.0 × 10^6^ nm^3^, 357 nm and 1.9 respectively. 

**Figure 4:**
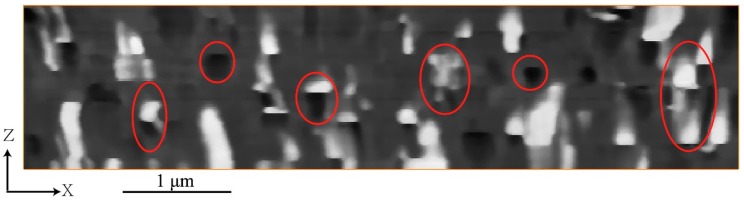
The 56th 2D image of the TiO_2_ pigmented waterborne paint film in the XZ direction. The grey parts are acrylic resin, the white parts are TiO_2_ particles, and the black parts are voids.

From the 2D images in Figure 1 and the 3D image in Figure 2, it can be clearly observed that there are many voids within the cured paint film. In Figure 2a and Figure 4, it can be observed that the voids are distributed in space in different forms. The XZ plane micrograph shown in Figure 4 is the image in the direction that is vertical to the XY plane from where the original BSE micrographs were obtained. The 2D image in XZ plane can show the relative position relationship and spatial distribution of voids and particles within the waterborne paint quite directly. From Figure 4, it can be clearly seen that there are many voids within the paint, some are isolated within the acrylic resin, some are connected with the TiO_2_ particles (in up-and-down direction), others are around the TiO_2_ particles, as presented in Figure 1 as well. Even in the raw data of Figure 4, there is a clear pattern of hemispherical particles on the outer side of the film, which end abruptly with a horizontal boundary and are immediately followed by a similarly shaped hemispherical void. We attribute these features to pigment particles escaping from the sample during the slicing by the diamond knife of the SBFSEM system—they are visible down to their waist, but invisible after the knife passes that point. This supports our following argument that the pigment particles are loosely bound to the paint matrix, probably in a quasi-liquid environment. This result has been reproduced on other paint samples, even well-aged and fully dried examples, suggesting that the surrounding liquid is a long-term configuration of the pigment.

**Figure 5:**
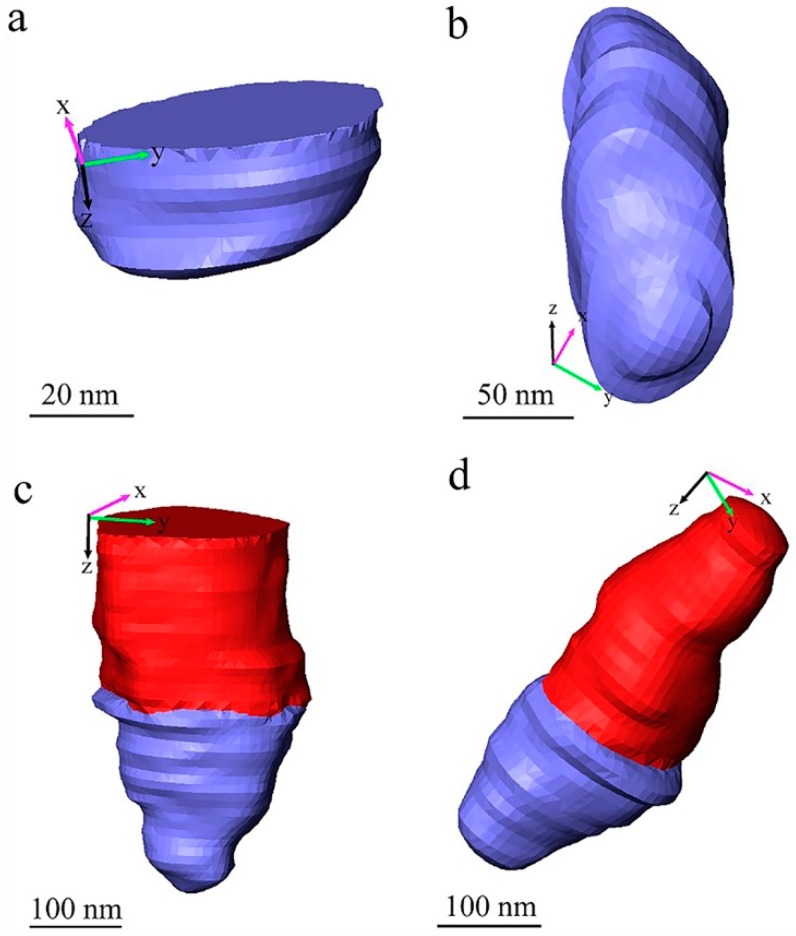
3D rendering of natural voids and voids produced by particle shedding in the TiO_2_ pigmented waterborne paint: (**a**) An incomplete natural void near the surface of the paint film; (**b**) An intact natural void inside the paint film; (**c**) A void produced by particle shedding connected to a TiO_2_ particle near the surface of the paint film; (**d**) Another void produced by particle shedding connected to a TiO_2_ particle inside the paint film. The red parts are TiO_2_ particles and the blue parts are voids.

**Figure 6:**
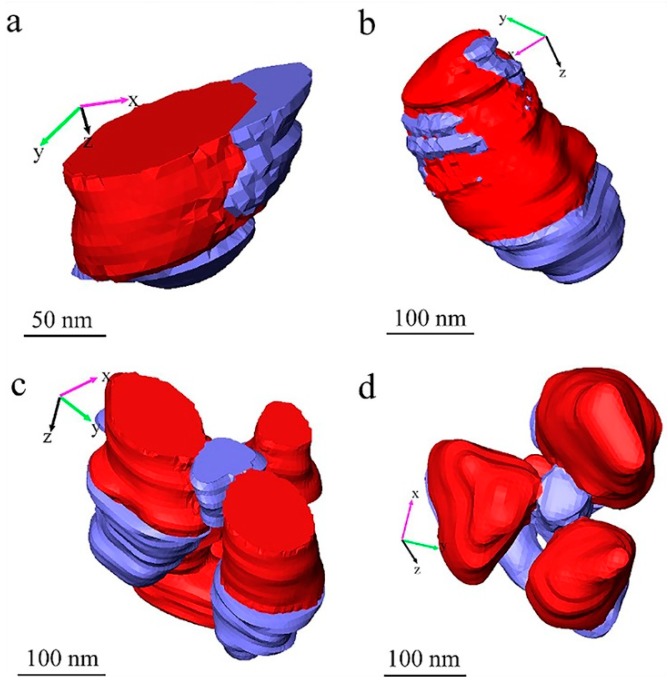
3D rendering of different voids in the TiO_2_ pigmented waterborne paint: (**a**) Voids around a TiO_2_ particle near the surface of the waterborne paint film; (**b**) Voids around a TiO_2_ particle in the waterborne paint film; (**c**) Voids around a tiny cluster of TiO_2_ particles near the surface of the waterborne paint film; (**d**) Voids around a tiny cluster of TiO_2_ particles in the waterborne paint film. The red parts are TiO_2_ particles and the blue parts are voids.

Figure 5 and Figure 6 show some typical voids in different forms in the measured sample in detail. With combined consideration of the images shown in Figure 1, Figure 2 and Figure 4, we propose that the forms of existence of the voids in the measured paint film can be classified into three types: voids that exist in the paint themselves, voids produced by particle shedding, and voids produced by quasi-liquid phase evaporation (during measurement). All the 3D images with voids in Figure 5 and Figure 6 were generated by manual processing using Avizo software (version 9.2, Thermo Fisher Scientific, Waltham, MA, USA), and they are either at the surface or inside the imaged volume of the paint film. As mentioned above, the blue parts are the imaged voids and the red parts are the TiO_2_ particles. In these 3D images in Figure 5 and Figure 6, the XY plane is the SEM imaging plane and the Z axis is the longitudinal imaging and sectioning direction which is perpendicular to the XY plane.

Figure 5a,b represent the isolated voids in the measured waterborne paint film—the void presented in Figure 5a is an incomplete void which exists near the surface of the reconstructed volume of the paint film; the void presented in Figure 5b is an intact void which is inside the paint film. The presence of such voids proves that there are natural voids in the dried paint film. These voids are very likely produced by the solvent evaporation during curing of the applied paint film. These voids play an important role in the appearance of the cured paint surface. 

Figure 5c,d represent the voids connected intimately with TiO_2_ particles, in which Figure 5c shows an incomplete TiO_2_ particle with the void located near the surface of the reconstructed paint film. From careful observation of the Supplementary Video S1 and Figure 5, there is a sharp boundary between the pigment particle and the void, aligned with the cutting direction. We conclude that these voids, seen directly in the raw image of Figure 4 as well, are produced by pulling the TiO_2_ particles out of the paint materials by the diamond knife cutting during SBFSEM imaging. It means that these kind of voids are actually cutting artefacts, not “real” pores in the cured paint film. This feature can be denoted particle “shedding” in this article. 

The statistical result from the 3D image segmentation obtained by Avizo gives out that the total volume of the TiO_2_ particles, the voids, and the acrylic resin are 5.793 µm^3^, 7.693 µm^3^ and 49.889 µm^3^, respectively, with a total analyzed paint volume of 63.375 µm^3^ as shown in Figure 2a. Since the densities of the TiO_2_ particles and the acrylic resin are 4.23 g/cm^3^ and 1.05 g/cm^3^, the measured weight fraction of the TiO_2_ particles is about 31% (density of TiO_2_ × volume of TiO_2_/(density of TiO_2_ × volume of TiO_2_ + density of acrylic resin × volume of acrylic resin)), which is only about four-fifths of the expected 38%. This result also supports our observation of the TiO_2_ particles shedding during SBFSEM measurement.

This TiO_2_ particle shedding was caused by the stiffness difference of the TiO_2_ pigment particles and the acrylic resin matrix and the loose of adhesion between these two materials during the sample’s constant exposure under electron beam illumination and continual mechanical cutting by the diamond knife. The electron beam illumination could break the chemical and/or physical bonding between the TiO_2_ pigment particles and the acrylic resin under the help from the electron-matter interactions and the vacuum environment in the SEM chamber, and then cause the matrix material lose adhesion with the pigment particles. With the help of the significant difference in the stiffness of these two materials, once the remaining parts of the TiO_2_ particles in the matrix materials became small enough and were disturbed by the external force from diamond knife cutting, the remaining parts of the TiO_2_ particles would then fall out of the acrylic resin matrix of the cured paint.

Figure 6 presents the third type of void, which are the voids around TiO_2_ particles. Figure 6a,c show the voids (in blue) near the surface of the measured volume of paint film; Figure 6b,d show the voids in the measured volume of paint film. In most of these cases in Figure 6, the TiO_2_ particles are finally falling out of the matrix materials, and then the second type of voids, the voids produced by particle shedding, are generated as well, as shown in all the examples displayed in Figure 6. Especially, in cases of (tiny) clusters of TiO_2_ particles as shown in Figure 6c,d, they are more likely to have voids around the TiO_2_ particles, which can be seen in Figure 1 as well. Considering the shedding of the TiO_2_ particles during the SBFSEM measurement, the formation of these kinds of voids around TiO_2_ particles in the cured paint film could be caused by evaporation of the liquid phase or moisture in the cured “dry” paint film. Since the specimen was cut and measured in the high vacuum system, the liquid phase in the specimen can be removed by the vacuum system of SBFSEM. As such, these voids are considered voids produced by quasi-liquid phase evaporation. This phenomenon indicates that, in the dry cured waterborne paint film, the TiO_2_ pigment particles are still in a quasi-liquid environment. This could be supported by the rational motion of nanoparticles found in viscous or viscoelastic medium under X-ray illumination [23] which also indicates the nanoparticles could be in a “non-solid” environment in materials normally considered as “solids”, such as paints [24]. It can be seen that the quasi-liquid phases in the cured waterborne paint film tend to appear around the TiO_2_ particles, especially when the TiO_2_ particles clustered. As such, the amount of quasi-liquid phases in the cured paint film would rise with the increase of the amount of TiO_2_ particles used and the number of TiO_2_ particle clusters. Therefore, as evident, if the clusters of the TiO_2_ particles in the cured paint film can be effectively reduced, the quasi-liquid phases or voids in the coating can be effectively reduced as well, and the demand for the TiO_2_ particles can also be reduced. Not can this help reduce the manufacturing cost of the paint, it can improve the physical and chemical performance of the cured paint.

**Figure 7:**
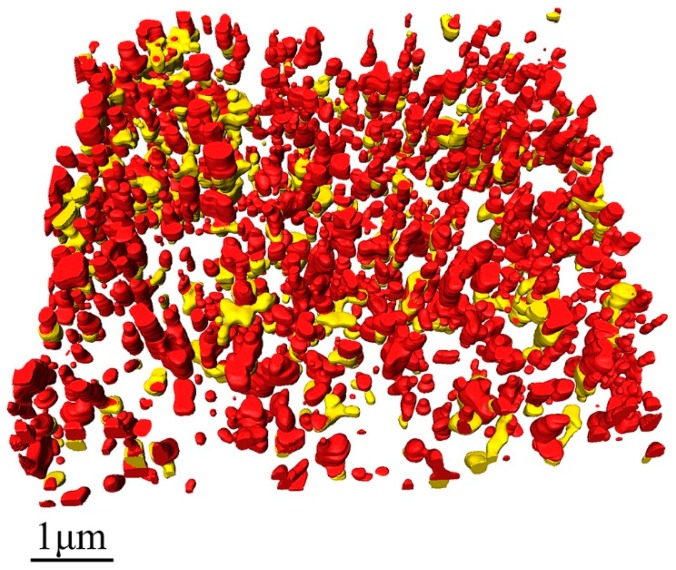
Rendering of the 3D spatial distribution of the TiO_2_ particles after correction. The red parts are the original TiO_2_ particles and the yellow parts are the shed parts of the TiO_2_ particles that were the voids caused by particle shedding before correction.

**Figure 8:**
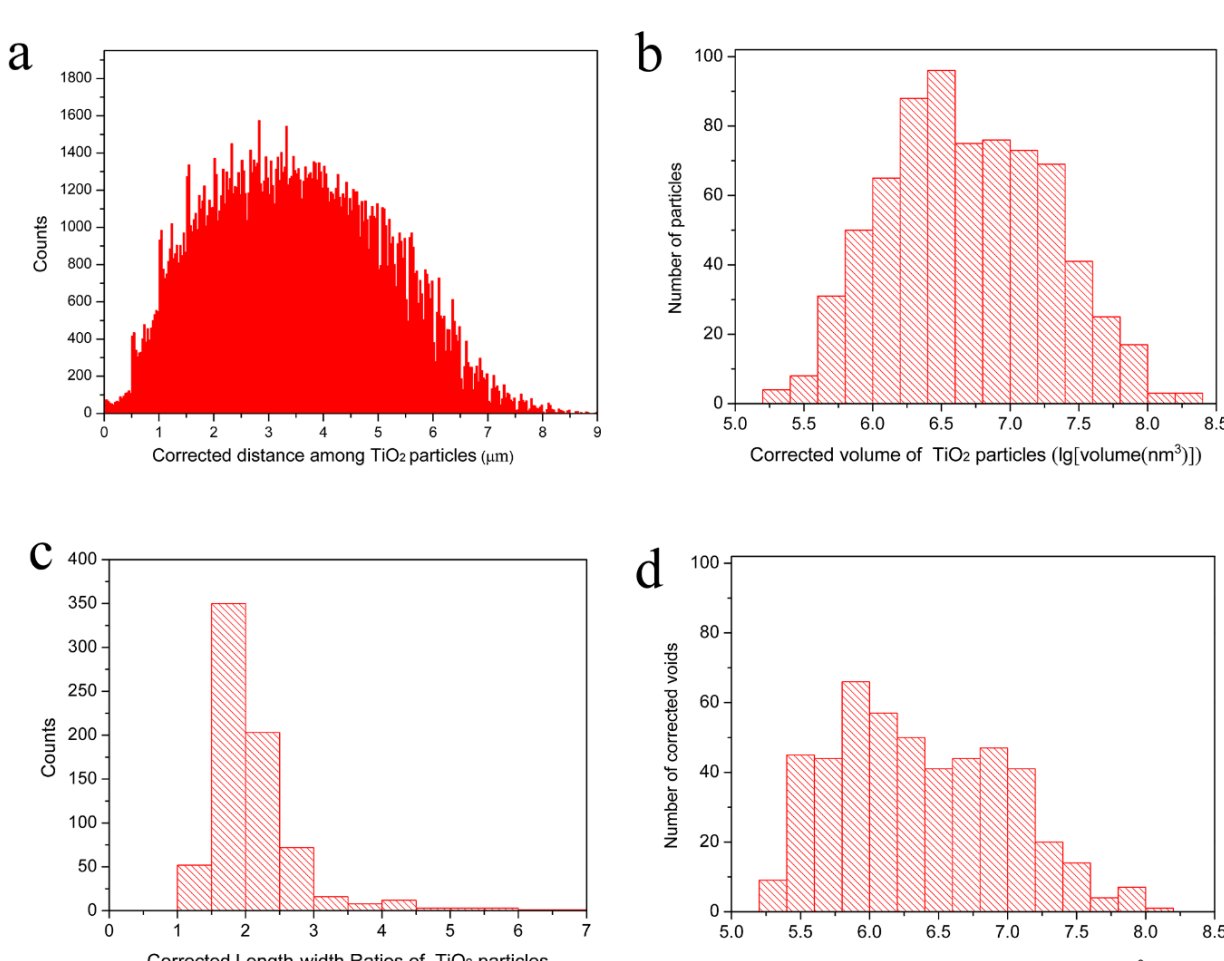
Corrected 3D characterization of the TiO_2_ particles and voids within the measured waterborne paint film: (**a**) A histogram of corrected distribution of distances among individual TiO_2_ particles; (**b**) A histogram of the corrected volume distribution of the TiO_2_ particles; (**c**) A histogram of the corrected length–width ratios of the measured TiO_2_ particles; (**d**) A histogram of the corrected volume distribution of the voids.

From the above discussion, we know that the second type of voids that connected intimately with TiO_2_ particles caused by particle shedding should be parts of the corresponding TiO_2_ particles connected with them. In order to eliminate the errors caused by this artefact, such as lowering the sizes and volumes of the TiO_2_ particles, we added the voxels of these voids to their connected TiO_2_ particles in the processed 3D image, i.e. these voids have been corrected and treated as parts of their connected TiO_2_ particles in the improved statistical analysis as presented in Figure 7 and Figure 8.

In Figure 7, the processed 3D image, in which the voids caused by particle shedding, were newly rendered as parts of TiO_2_ particles connected with them (on the basis of Figure 2b). The statistical analysis results after adding the voids caused by particle shedding to their connected TiO_2_ particles are shown in Figure 8. The corrected distribution of the distances among different TiO_2_ particles are clearly presented in Figure 8a, it can be known that most of the distances, about 76%, are still between 1.5 µm and 5.5 µm and they have a nearly Gaussian distribution as well, which is almost the same as shown in Figure 3a. However, the minimum distance between the particles becomes 93 nm, which is shorter than 124 nm before correction. This is caused by the corrected TiO_2_ particles that are larger and occupy more spaces in the measured volume. The corrected volumes of the single TiO_2_ particles displayed in Figure 8b range from 2.0 × 10^5^ nm^3^ to 2.0 × 10^8^ nm^3^. More than 55% of the measured TiO_2_ particles have volumes between 1.0 × 10^6^ nm^3^ and 1.0 × 10^7^ nm^3^. The volume of the largest TiO_2_ particles (2.0 × 10^8^ nm^3^) after correction is about twice as that of the largest TiO_2_ particles (8.7 × 10^7^ nm^3^) before correction, which indicates that the largest TiO_2_ particles tend to be shed at the half way during the cutting-imaging process by SBFSEM. The corrected length–width ratios of the TiO_2_ particles presented in Figure 8c are between 1.3 and 6.5, and 84% of them are between 1.3 and 2.5. The corrected volume of the voids in the paint presented in Figure 8d shows that volume of the individual voids is between 1.8 × 10^5^ nm^3^ and 1.3 × 10^8^ nm^3^, the same as before correction, but the quantity of voids after correction is about two thirds of that before correction. Yet, there has not been a great change in the volume distribution of the voids after correction. 67% of the voids are between 1.0 × 10^6^ nm^3^ and 1.0 × 10^8^ nm^3^, only slightly lower than 74% before correction. It can also be obtained that the average volume, length, and length–width ratios of the corrected TiO_2_ particles are 1.2 × 10^7^ nm^3^, 396 nm, and 2.1 that are larger than 8.0 × 10^6^ nm^3^, 357 nm, and 1.9 before correction. This can be easily understood because the corrected TiO_2_ particles are larger and longer than before. 

After correction, the total volume of TiO_2_ particles increases from 5.793 µm^3^ to 7.838 µm^3^, so the measured weight fraction of the TiO_2_ particles increases from 31% to 38.3%, which is very close to the expected 38%. This result turns out again supporting that the TiO_2_ particles are shedding during SBFSEM measurement, and SBFSEM is proven to be a reliable tool for 3D nano-structure investigation for materials.

## 4. Conclusions

SBFSEM method provides high resolution and enables the nanoscale details of the TiO_2_ particles pigmented waterborne paint to be revealed, although the fresh surfaces of the sample imaged by the electron microscope are produced by mechanically cutting using an ultra-microtome by diamond knife. The method is physically destructive and may cause deformation of the internal structure of the specimen such as the falling off of the TiO_2_ pigment particles from the paint sample during measurement.

The 3D spatial structure of the TiO_2_ particle pigmented waterborne paint was revealed and analyzed. The TiO_2_ particles are rice-shaped and have a preferred volume range between 2.0 × 10^5^ nm^3^ and 2.0 × 10^8^ nm^3^. The average volume, length, and length–width ratios of the corrected TiO_2_ particles are 1.2 × 10^7^ nm^3^, 396 nm and 2.1 respectively and the corrected minimum distance between different particles is 93 nm. Three different types of voids can be found in the 3D image of the measured volume of the cured waterborne paint film: voids that exist in the paint themselves, voids produced by particle shedding, and voids produced by quasi-liquid phase evaporation. Among them, the voids produced by particle shedding are cutting artefacts and the voids produced by quasi-liquid phase evaporation are a kind of damage caused by high vacuum environment and electron beam illumination to the sample itself. 

The results will help to understand the real 3D spatial structure of waterborne paints, especially the 3D spatial distribution of TiO_2_ particles in the cured paint film. The work also provide evidence to support that the pigment particles could be in a quasi-liquid environment in the “solid” cured paint film. The results will lay a base for a better understanding of the detailed spatial structure of paints and coatings.

## Supplementary Materials

The following supplementary materials are available online at http://www.mdpi.com/1996-1944/12/3/464/s1, Table S1: Length, width, volume and length–width ratio of TiO_2_ particles; Video S1: Aligned serial slice images of the TiO_2_ containing waterborne paint.

## References

[1] Nguyen T.V., Tri P.N., Nguyen T.D., El Aidani R., Trinh V.T., Decker C. (2016). Accelerated degradation of water borne acrylic nanocomposites used in outdoor protective coatings. Polym. Degrad. Stabil..

[2] Akbarian M., Olya M.E., Mahdavian M., Ataeefard M. (2014). Effects of nanoparticulate silver on the corrosion protection performance of polyurethane coatings on mild steel in sodium chloride solution. Prog. Org. Coat..

[3] Anees S.M., Dasari A. (2017). A review on the environmental durability of intumescent coatings for steels. J. Mater. Sci..

[4] Weiss K.D. (1997). Paint and coatings: A mature industry in transition. Prog. Polym. Sci..

[5] Castillo L.A., Barbosa S.E., Maiza P., Capiati N.J. (2010). Surface modifications of talcs. Effects of inorganic and organic acid treatments. J. Mater. Sci..

[6] Nasir K.M., Sulong N.H.R., Johan M.R., Afifi A.M. (2017). An investigation into waterborne intumescent coating with different fillers for steel application. Pigment Resin Technol..

[7] Noreen A., Zia K.M., Zuber M., Tabasum S., Saif M.J. (2015). Recent trends in environmentally friendly water-borne polyurethane coatings: A review. Korean J. Chem. Eng..

[8] Karakas F., Celik M.S. (2012). Effect of quantity and size distribution of calcite filler on the quality of water borne paints. Prog. Org. Coat..

[9] Wojciechowski K., Zukowska G.Z., Korczagin I., Malanowski P. (2015). Effect of TiO_2_ on UV stability of polymeric binder films used in waterborne facade paints. Prog. Org. Coat..

[10] Farrokhpay S., Morris G.E., Fornasiero D. (2006). Titania Pigment Particles Dispersion in Water-Based Paint Films. JCT Res..

[11] Vallittu P.K. (1999). Flexural properties of acrylic resin polymers reinforced with unidirectional and woven glass fibers. J. Prosthet. Dent..

[12] Karakas F., Hassas B.V., Celik M.S. (2015). Effect of precipitated calcium carbonate additions on waterborne paints at different pigment volume concentrations. Prog. Org. Coat..

[13] Denk W., Horstmann H. (2004). Serial block-face scanning electron microscopy to reconstruct three-dimensional tissue nanostructure. PLoS Biol..

[14] Leighton S.B. (1981). SEM images of block faces, cut by a miniature microtome within the SEM–a technical note. Scan Electron. Microsc..

[15] Hashimoto T., Thompson G.E., Zhou X., Withers P.J. (2016). 3D imaging by serial block face scanning electron microscopy for materials science using ultramicrotomy. Ultramicroscopy.

[16] Chen B., Yusuf M., Hashimoto T., Estandarte A.K., Thompson G., Robinson I. (2017). Three-dimensional positioning and structure of chromosomes in a human prophase nucleus. Sci. Adv..

[17] Arenkiel B.R., Ehlers M.D. (2009). Molecular genetics and imaging technologies for circuit-based neuroanatomy. Nature.

[18] Lipke E., Hornschemeyer T., Pakzad A., Booth C.R., Michalik P. (2014). Serial block-face imaging and its potential for reconstructing diminutive cell systems: A case study from arthropods. Microsc. Microanal..

[19] Chen B., Guizar-Sicairos M., Xiong G., Shemilt L., Diaz A., Nutter J., Burdet N., Huo S., Mancuso J., Monteith A. (2013). Three-dimensional structure analysis and percolation properties of a barrier marine coating. Sci. Rep..

[20] Thompson G.E., Hashimoto T., Zhong X.L., Curioni M., Zhou X., Skeldon P., Withers P.J., Carr J.A., Monteith A.G. (2013). Revealing the three dimensional internal structure of aluminium alloys. Surf. Interface Anal..

[21] Chen B., Hashimoto T., Vergeer F., Burgess A., Thompson G., Robinson I. (2014). Three-dimensional analysis of the spatial distribution of iron oxide particles in a decorative coating by electron microscopic imaging. Prog. Org. Coat..

[22] Barbieri D., Yuan H., Ismailoglu A.S., de Bruijn J.D. (2017). Comparison of two moldable calcium phosphate-based bone graft materials in a noninstrumented canine interspinous implantation model. Tissue Eng. Part..

[23] Liang M.N., Harder R., Robinson I. (2014). Brownian motion studies of viscoelastic colloidal gels by rotational single particle tracking. IUCrJ.

[24] Liang M.N., Harder R., Robinson I. (2018). Radiation-driven rotational motion of nanoparticles. J. Synchrotron Radiat..

