# Supplementary material for: Investigation of Three-Dimensional Structure and Pigment Surrounding Environment of a TiO2 Containing Waterborne Paint
Source: Materials (Basel). 2019 Feb 2;12(3):464. doi: 10.3390/ma12030464 (PMC6384949; doi:10.3390/ma12030464)
Supplement: Supplementary file 1 [file materials-12-00464-s001.zip › Table S1.pdf]

materials

MDPI

Table S1. Length, width, volume and length-width ratio of TiO_2 particles.

Number	Length (nm)	Width (nm)	Volume (nm^3)	Length-width ratio
1	411	224	10185600	1.84
2	140	69	395460	2.03
3	377	187	6958580	2.02
4	286	145	3143400	1.97
5	110	66	253500	1.66
6	817	338	43084900	2.42
7	238	112	1716200	2.13
8	124	67	321945	1.84
9	377	225	8256500	1.67
10	692	360	33963900	1.92
11	367	165	4720170	2.23
12	552	216	8461830	2.56
13	498	206	9275560	2.42
14	566	332	20358600	1.71
15	429	234	13369600	1.84
16	339	147	3237200	2.31
17	326	171	4007840	1.91
18	412	224	11313700	1.84
19	306	133	2301780	2.29
20	543	271	19915000	2.01
21	315	221	7341360	1.43
22	330	216	6900270	1.53
23	110	51	228150	2.14
24	404	281	14114900	1.44
25	640	386	22546300	1.66
26	622	273	17483900	2.28
27	466	249	16938900	1.88
28	326	227	7009280	1.43
29	266	166	3658000	1.60
30	468	236	9638070	1.98
31	437	276	16333000	1.59
32	362	213	6966180	1.70
33	335	210	6532700	1.59
34	396	227	10621600	1.75
35	241	106	1140750	2.28
36	392	247	13554600	1.58

37	205	62	973440	3.33
38	454	191	10238900	2.37
39	566	199	9166560	2.84
40	397	206	9516390	1.93
41	294	177	4007840	1.66
42	641	245	14672600	2.62
43	345	161	4821570	2.15
44	432	245	13151600	1.76
45	1030	396	47290400	2.60
46	230	142	2142080	1.62
47	578	206	7850900	2.80
48	614	244	17904700	2.51
49	866	380	42253400	2.28
50	291	184	5163800	1.58
51	334	214	6611280	1.56
52	637	207	10877700	3.07
53	353	232	10892900	1.52
54	268	155	3123120	1.73
55	295	157	4139660	1.88
56	547	226	13914600	2.42
57	400	182	5901480	2.19
58	305	201	5663190	1.52
59	387	179	7176580	2.17
60	424	261	12023500	1.62
61	214	155	2050820	1.38
62	237	93	1579300	2.55
63	1013	389	39082100	2.61
64	647	229	12487400	2.82
65	479	240	11260500	1.99
66	560	212	12076700	2.64
67	608	276	20069600	2.20
68	281	166	3614910	1.69
69	392	219	8114540	1.79
70	244	158	2740340	1.55
71	282	167	4684680	1.69
72	654	307	27763300	2.13
73	185	115	1244680	1.61
74	542	257	13823400	2.11
75	558	213	20150700	2.62
76	347	214	6063720	1.62

77	319	210	5473060	1.52
78	187	99	661635	1.89
79	340	210	6558040	1.62
80	368	160	5746840	2.30
81	301	173	6195540	1.74
82	546	257	16295000	2.13
83	327	174	5090280	1.88
84	1240	510	74478300	2.43
85	517	320	21266100	1.62
86	301	149	2235870	2.02
87	260	168	2755540	1.55
88	626	256	25996400	2.45
89	391	240	9409920	1.63
90	230	143	1898720	1.62
91	251	119	1541280	2.11
92	969	497	87632400	1.95
93	253	161	3204240	1.57
94	317	169	5407160	1.88
95	466	193	12403800	2.42
96	243	158	2367690	1.54
97	426	258	12228800	1.65
98	485	296	12687700	1.64
99	685	351	33543100	1.95
100	333	184	6279200	1.81
101	100	51	190125	1.96
102	540	262	18034000	2.06
103	813	355	32359300	2.29
104	516	263	16287400	1.96
105	377	239	11529200	1.58
106	663	229	14659900	2.89
107	199	60	646425	3.31
108	311	165	3579420	1.88
109	320	122	2590770	2.61
110	386	167	7711470	2.31
111	329	139	3584490	2.37
112	304	172	3952060	1.76
113	746	193	14104700	3.87
114	290	145	3526180	2.00
115	277	150	2953280	1.85
116	211	111	1145820	1.90

117	327	183	5285480	1.78
118	566	285	13787900	1.99
119	494	196	13610400	2.52
120	255	171	3148470	1.49
121	235	124	1556490	1.90
122	297	202	4786080	1.47
123	198	127	1627470	1.56
124	537	146	5166330	3.68
125	236	159	2347410	1.49
126	276	166	2915250	1.67
127	282	152	2702310	1.86
128	408	190	6076400	2.15
129	191	105	861900	1.81
130	276	162	4225840	1.70
131	578	234	14708100	2.47
132	178	103	747825	1.73
133	210	123	1472840	1.71
134	230	153	2311920	1.50
135	159	100	656565	1.60
136	450	241	12269400	1.87
137	186	107	836550	1.75
138	538	250	12327700	2.15
139	398	165	6854640	2.41
140	618	210	10915700	2.94
141	136	60	387855	2.26
142	327	189	3876020	1.74
143	649	245	15344400	2.65
144	350	202	7772310	1.73
145	515	303	13496300	1.70
146	236	171	2570490	1.38
147	454	242	9638070	1.88
148	232	151	2387970	1.53
149	557	196	12330200	2.84
150	563	299	21940400	1.88
151	508	294	16693000	1.73
152	536	205	11235100	2.61
153	192	134	1401860	1.44
154	621	314	23758000	1.97
155	354	156	3559140	2.27
156	1358	416	72757000	3.27

157	305	191	4836780	1.60
158	231	122	1863220	1.90
159	271	157	2793570	1.73
160	630	342	22559000	1.84
161	429	186	6717750	2.31
162	181	111	1031740	1.64
163	316	187	5860920	1.69
164	280	139	2377830	2.02
165	618	277	11181900	2.23
166	290	185	4443860	1.56
167	457	271	14056600	1.68
168	230	154	2322060	1.49
169	349	248	9447940	1.40
170	526	257	16974400	2.05
171	321	211	6920550	1.53
172	1072	560	72394500	1.91
173	451	256	10309800	1.76
174	832	482	46306800	1.73
175	747	276	22627400	2.71
176	211	139	1609720	1.52
177	319	184	5369130	1.74
178	818	465	35195900	1.76
179	175	111	826410	1.58
180	583	193	9610180	3.02
181	142	92	494325	1.54
182	150	96	656565	1.56
183	872	290	24295400	3.00
184	156	97	684450	1.61
185	280	155	2884830	1.80
186	532	291	12010800	1.83
187	177	101	899925	1.75
188	237	151	2246010	1.57
189	285	141	3774620	2.03
190	119	81	316875	1.47
191	399	187	7128420	2.13
192	272	193	3974880	1.41
193	321	151	3039460	2.12
194	121	76	291525	1.59
195	940	591	52132300	1.59
196	119	81	316875	1.47

197	289	137	2210520	2.11
198	280	131	3262540	2.14
199	309	136	2175030	2.28
200	276	146	2699780	1.89
201	584	309	28655600	1.89
202	119	81	316875	1.47
203	156	97	684450	1.61
204	545	274	15050300	1.99
205	860	282	24944400	3.05
206	557	175	6007950	3.18
207	150	99	654030	1.52
208	197	146	1817600	1.35
209	289	162	3194100	1.79
210	494	258	14160500	1.91
211	424	226	7764700	1.88
212	425	281	12464600	1.51
213	246	173	3285360	1.42
214	494	294	21623600	1.68
215	573	374	28544100	1.53
216	320	195	4550320	1.64
217	192	141	1607190	1.36
218	1124	436	51092900	2.58
219	593	251	13291000	2.37
220	246	149	2192780	1.65
221	530	294	22495600	1.80
222	315	215	7242500	1.47
223	903	459	31243900	1.97
224	275	193	4017980	1.43
225	224	125	1751680	1.79
226	179	124	1150890	1.45
227	159	111	821340	1.43
228	330	195	4748060	1.70
229	283	206	4846920	1.37
230	284	180	3876020	1.57
231	432	239	8269170	1.80
232	134	84	443625	1.60
233	258	157	2600910	1.64
234	212	150	2045740	1.41
235	119	81	316875	1.47
236	336	150	3627580	2.24

237	135	82	430950	1.64
238	333	177	3893760	1.88
239	598	283	16472400	2.11
240	192	115	1183840	1.67
241	257	159	2466560	1.62
242	443	263	16660000	1.69
243	198	115	1237080	1.72
244	165	97	732615	1.69
245	366	189	5810220	1.93
246	243	139	1886040	1.75
247	250	138	2271360	1.82
248	515	251	10261700	2.06
249	300	150	2960880	2.01
250	185	110	963300	1.68
251	377	211	7972580	1.79
252	162	81	633750	2.00
253	420	199	9524000	2.11
254	437	222	10583600	1.97
255	416	199	5696140	2.09
256	185	104	823875	1.78
257	336	132	2040680	2.54
258	210	119	1450020	1.76
259	413	235	6601140	1.76
260	329	192	6715220	1.71
261	343	248	7954830	1.39
262	226	146	1957020	1.54
263	317	144	2476700	2.20
264	427	174	4791150	2.46
265	274	123	1979840	2.22
266	373	175	4071210	2.13
267	339	208	5680940	1.63
268	231	138	2922860	1.68
269	196	128	1310600	1.53
270	375	185	3881080	2.02
271	690	346	24556500	2.00
272	410	233	8385780	1.76
273	371	223	8565760	1.67
274	462	268	11673700	1.72
275	313	169	3632660	1.85
276	194	108	823875	1.80

277	151	98	557700	1.53
278	684	365	14497700	1.87
279	1326	670	86846600	1.98
280	452	213	10895400	2.12
281	491	201	7673440	2.45
282	226	160	2349940	1.42
283	308	194	4882410	1.59
284	149	97	608400	1.53
285	167	100	887250	1.67
286	258	120	2225730	2.15
287	194	112	1338480	1.73
288	188	103	1133140	1.83
289	538	214	13377200	2.52
290	527	272	12515300	1.94
291	135	89	443625	1.51
292	384	120	2329660	3.20
293	565	239	13805600	2.36
294	377	171	6563120	2.21
295	287	158	3196640	1.82
296	721	364	20761600	1.98
297	342	196	5805150	1.74
298	235	125	2028000	1.88
299	267	160	3569280	1.66
300	711	370	43526000	1.92
301	394	286	12705400	1.38
302	177	120	1188920	1.48
303	161	96	709800	1.68
304	717	413	44524700	1.73
305	176	127	1064700	1.39
306	330	194	5825430	1.70
307	133	82	443625	1.62
308	245	130	2197840	1.89
309	851	237	25342400	3.59
310	236	150	2671890	1.57
311	176	114	1026680	1.54
312	144	86	507000	1.68
313	305	212	5217030	1.44
314	362	227	7752030	1.60
315	248	145	3019180	1.71
316	464	224	9037280	2.07

317	425	245	8124680	1.73
318	280	118	1619860	2.37
319	248	149	2476700	1.67
320	1049	576	70850700	1.82
321	138	86	443625	1.61
322	587	319	23605900	1.84
323	132	57	296595	2.33
324	175	95	747825	1.85
325	328	208	4560460	1.58
326	144	92	519675	1.56
327	338	193	5255060	1.75
328	217	123	1848020	1.77
329	119	81	316875	1.47
330	177	110	937950	1.62
331	242	148	2139540	1.63
332	526	220	9336400	2.40
333	589	308	14218800	1.91
334	183	115	950625	1.59
335	167	104	707265	1.60
336	119	81	316875	1.47
337	105	61	190125	1.72
338	468	275	12256700	1.70
339	206	140	1604660	1.47
340	156	113	811200	1.39
341	364	164	5120700	2.21
342	418	182	5528840	2.29
343	143	97	593190	1.47
344	938	557	52697600	1.68
345	327	188	6066260	1.74
346	143	90	507000	1.59
347	518	185	5391940	2.79
348	154	88	570375	1.75
349	271	188	4106700	1.44
350	266	188	3914040	1.42
351	478	285	15042700	1.68
352	339	194	5645440	1.75
353	930	296	43074700	3.15
354	119	81	316875	1.47
355	183	122	1084980	1.50
356	197	112	998790	1.75

357	225	171	2829060	1.32
358	147	84	545025	1.76
359	521	173	6950970	3.01
360	155	99	666705	1.57
361	609	293	20386500	2.08
362	349	159	3751800	2.20
363	352	133	2823990	2.65
364	612	283	11990600	2.16
365	305	168	4078820	1.82
366	189	136	1452560	1.39
367	438	258	10013200	1.69
368	603	298	16193600	2.02
369	323	124	2676960	2.61
370	268	151	2763150	1.77
371	259	116	2849340	2.23
372	540	293	21108900	1.84
373	614	280	24460200	2.19
374	498	275	13782800	1.81
375	592	276	20688100	2.14
376	507	192	7343900	2.64
377	306	197	4664400	1.55
378	305	204	4149800	1.50
379	321	197	5156190	1.63
380	119	81	316875	1.47
381	280	145	2780900	1.93
382	119	81	316875	1.47
383	171	97	927810	1.76
384	692	256	14637100	2.70
385	592	281	16300000	2.11
386	339	213	4504700	1.59
387	163	85	684450	1.91
388	564	279	21070900	2.02
389	389	219	7115740	1.78
390	715	370	15795600	1.93
391	273	165	3176360	1.66
392	404	199	8918130	2.03
393	943	414	53161500	2.28
394	205	131	1754220	1.56
395	857	366	35652200	2.34
396	334	168	4737920	1.99

397	1006	439	54259100	2.29
398	684	237	13017200	2.89
399	117	64	228150	1.82
400	616	192	12609100	3.20
401	179	120	1059630	1.49
402	277	171	3779680	1.62
403	315	189	4849460	1.67
404	309	190	4304430	1.63
405	284	170	4507230	1.67
406	262	176	3908970	1.49
407	585	221	15767700	2.65
408	984	366	37885600	2.69
409	520	220	9384570	2.36
410	446	258	10908100	1.73
411	343	171	5199280	2.00
412	788	333	22084900	2.37
413	150	99	583050	1.52
414	667	280	24140800	2.38
415	181	112	1031740	1.62
416	243	165	2780900	1.47
417	331	194	6692400	1.71
418	228	161	2631330	1.41
419	554	210	7252640	2.64
420	439	206	8456760	2.13
421	723	304	26374100	2.38
422	348	166	5680940	2.09
423	227	126	1766900	1.80
424	824	459	40788200	1.80
425	352	201	8852220	1.75
426	226	135	1774500	1.68
427	244	119	1989980	2.05
428	221	120	1498180	1.84
429	275	158	2803710	1.74
430	674	278	16822300	2.43
431	402	221	7802730	1.82
432	562	280	11835900	2.00
433	752	232	12713000	3.24
434	186	99	996255	1.89
435	239	160	2504580	1.49
436	496	158	6018090	3.14

437	611	286	21768000	2.14
438	310	174	4357660	1.78
439	196	118	1399320	1.66
440	229	162	2418390	1.42
441	166	102	732615	1.63
442	433	213	7830620	2.04
443	435	261	11184400	1.67
444	238	178	3168750	1.34
445	475	274	9288240	1.74
446	678	338	21973400	2.00
447	584	198	10535500	2.95
448	256	177	3384220	1.45
449	626	330	27208200	1.90
450	274	167	3019180	1.64
451	206	145	1908860	1.42
452	233	131	1984900	1.79
453	149	95	545025	1.58
454	182	99	922740	1.85
455	1028	675	82795600	1.52
456	528	176	5942040	2.99
457	705	366	33398600	1.93
458	381	226	9498640	1.69
459	119	81	316875	1.47
460	195	133	1267500	1.47
461	890	307	29515000	2.90
462	488	170	6956040	2.87
463	643	304	20523400	2.11
464	128	83	380250	1.54
465	205	96	849225	2.13
466	587	255	16436900	2.30
467	356	201	4928040	1.77
468	671	267	33870100	2.52
469	315	177	5133380	1.78
470	242	160	2976090	1.52
471	222	149	2038140	1.49
472	248	136	3224520	1.82
473	986	663	64799700	1.49
474	123	76	329550	1.60
475	748	337	26582000	2.22
476	235	139	1809990	1.69

477	664	260	18317900	2.55
478	219	149	2261220	1.47
479	276	164	2887360	1.68
480	196	128	1559020	1.53
481	680	326	17843900	2.08
482	447	200	8560700	2.23
483	274	177	3470420	1.55
484	793	310	18786900	2.56
485	194	120	1356220	1.61
486	434	226	5450250	1.92
487	158	95	621075	1.68
488	325	203	4679610	1.60
489	581	344	21240800	1.69
490	254	123	1541280	2.07
491	328	221	5315900	1.49
492	164	112	922740	1.46
493	197	146	1995040	1.35
494	176	69	517140	2.54
495	284	171	3876020	1.66
496	335	150	2504580	2.24
497	190	114	1242150	1.68
498	119	81	316875	1.47
499	188	128	1480440	1.46
500	173	123	1176240	1.41
501	571	238	15395100	2.40
502	832	461	57017200	1.80
503	173	111	851760	1.56
504	633	253	19182300	2.50
505	185	110	1014000	1.68
506	482	195	9480900	2.47
507	299	153	2867080	1.96
508	237	161	2714980	1.47
509	119	81	316875	1.47
510	546	282	17686700	1.94
511	593	193	8289450	3.07
512	275	167	3227060	1.65
513	309	182	4585820	1.70
514	684	288	22837800	2.38
515	586	236	10682500	2.48
516	464	152	5721500	3.06

517	210	111	1183840	1.89
518	594	264	12086900	2.25
519	199	143	1739010	1.39
520	176	114	1039350	1.55
521	207	138	1662960	1.50
522	491	263	15331700	1.87
523	315	175	5369130	1.80
524	256	135	2458950	1.90
525	207	107	1335940	1.94
526	291	134	3399440	2.18
527	173	100	897390	1.73
528	189	128	1330880	1.48
529	410	246	11050100	1.67
530	522	261	12738400	2.00
531	203	135	1749150	1.50
532	596	264	11377100	2.25
533	166	111	879645	1.50
534	106	55	190125	1.93
535	166	110	844155	1.50
536	158	98	656565	1.60
537	544	197	6885060	2.77
538	646	274	16497800	2.36
539	738	431	27182800	1.71
540	315	177	4377940	1.78
541	484	212	9911850	2.28
542	434	228	12363200	1.90
543	380	215	9818060	1.77
544	365	151	5034510	2.42
545	377	182	5255060	2.07
546	122	57	266175	2.14
547	205	129	1498180	1.59
548	361	190	5914160	1.90
549	216	129	1513400	1.67
550	323	150	2730200	2.16
551	346	199	6372990	1.74
552	119	81	316875	1.47
553	494	241	9331340	2.05
554	445	188	5336180	2.38
555	346	161	5077600	2.15
556	499	322	24138300	1.55

557	320	143	2441200	2.23
558	198	111	1059630	1.78
559	221	152	1984900	1.46
560	244	162	3064820	1.50
561	531	184	9136140	2.89
562	282	162	3480560	1.74
563	376	193	6375520	1.94
564	534	325	16875500	1.64
565	448	244	13144000	1.83
566	244	169	2930460	1.44
567	215	142	1926600	1.51
568	183	128	1275100	1.42
569	404	228	10046200	1.77
570	319	151	4185280	2.11
571	540	265	12236400	2.04
572	242	151	3300570	1.60
573	445	230	12604000	1.93
574	312	163	4388080	1.92
575	344	171	5004090	2.01
576	559	358	16249400	1.56
577	386	196	6760840	1.97
578	282	181	4233450	1.55
579	433	217	8613930	2.00
580	161	104	735150	1.55
581	306	142	2476700	2.15
582	488	233	10783900	2.09
583	299	214	5736700	1.39
584	498	221	7136020	2.26
585	426	190	7143630	2.24
586	292	165	3376620	1.77
587	408	216	8114540	1.89
588	119	81	316875	1.47
589	223	126	1708590	1.76
590	187	95	793455	1.97
591	639	347	20467600	1.84
592	263	152	2661750	1.73
593	181	124	1090050	1.46
594	143	99	580515	1.45
595	364	128	2813850	2.85
596	440	263	9191910	1.67

597	373	186	7229820	2.00
598	369	163	3576880	2.26
599	356	194	7432620	1.83
600	229	131	1673100	1.74
601	240	113	1510860	2.12
602	248	142	2745400	1.74
603	141	97	570375	1.45
604	222	139	2253620	1.60
605	732	266	14398800	2.75
606	255	175	3475480	1.45
607	316	197	5042120	1.61
608	149	97	557700	1.53
609	228	145	1766900	1.58
610	718	284	24622500	2.53
611	292	111	1870830	2.65
612	388	227	6045980	1.71
613	196	114	1381580	1.72
614	316	212	5959780	1.49
615	382	173	6279200	2.21
616	197	143	1632540	1.38
617	334	145	3878550	2.31
618	252	189	4471740	1.33
619	174	110	856830	1.59
620	144	85	494325	1.70
621	255	138	1972230	1.85
622	532	176	6948440	3.03
623	327	157	4167540	2.08
624	284	152	2968480	1.87
625	238	163	2841740	1.46
626	324	130	3102840	2.50
627	106	55	190125	1.93
628	156	101	656565	1.55
629	409	236	9828200	1.74
630	196	130	1399320	1.51
631	307	135	4210640	2.27
632	285	185	4106700	1.54
633	268	142	2088840	1.89
634	372	212	6129630	1.75
635	255	133	1962090	1.91
636	391	235	8221000	1.66

637	354	217	6570720	1.63
638	464	235	8510000	1.97
639	363	162	3914040	2.25
640	258	170	2978620	1.52
641	268	164	3062280	1.64
642	582	217	10753500	2.68
643	250	191	3500840	1.31
644	295	200	4535120	1.48
645	472	204	7937080	2.32
646	280	141	2971020	1.99
647	261	151	2319520	1.72
648	132	83	418275	1.59
649	135	82	430950	1.64
650	140	84	494325	1.68
651	257	183	3452670	1.40
652	196	130	1376500	1.51
653	255	122	2106580	2.09
654	160	109	811200	1.47
655	175	121	1026680	1.45
656	262	118	2405720	2.23
657	262	192	4235980	1.36
658	262	147	2322060	1.78
659	119	81	316875	1.47
660	355	184	4841850	1.94
661	233	145	2631330	1.61
662	226	132	1566630	1.71
663	244	164	2603440	1.49
664	231	147	2091380	1.58
665	219	141	1901250	1.55
666	358	167	4884940	2.14
667	202	131	1432280	1.54
668	199	148	1561560	1.34
669	192	130	1333410	1.48
670	269	141	2306850	1.91
671	267	131	3199170	2.04
672	209	132	2119260	1.58
673	317	139	4215700	2.28
674	199	95	1204120	2.10
675	183	125	1095120	1.46
676	180	96	836550	1.88

677	177	115	1074840	1.54
678	250	115	1728870	2.18
679	335	128	3645330	2.62
680	173	111	988650	1.56
681	204	114	1432280	1.80
682	241	112	1455090	2.15
683	316	125	3924180	2.52
684	266	119	2583160	2.24
685	495	128	10195800	3.87
686	194	115	1574240	1.69
687	172	115	1363830	1.50
688	182	112	1199060	1.63
689	285	123	3183960	2.32
690	232	115	1903780	2.02
691	167	99	920205	1.70
692	153	97	699660	1.57
693	295	101	2251080	2.92
694	180	97	1019070	1.85
695	360	108	3156080	3.34
696	257	110	2192780	2.33
697	180	97	1014000	1.85
698	230	108	2012790	2.14
699	236	97	1574240	2.43
700	187	99	1219340	1.90
701	133	81	456300	1.64
702	119	81	316875	1.47
703	183	82	1039350	2.22
704	126	81	405600	1.55
705	180	82	719940	2.18
706	147	82	529815	1.78
707	125	81	367575	1.55
708	180	86	1077380	2.08
709	255	95	2251080	2.68
710	155	82	580515	1.88
711	117	66	291525	1.76
712	125	66	329550	1.89
713	190	70	846690	2.71
714	132	67	400530	1.95
715	128	67	359970	1.89
716	173	70	628680	2.47

717	153	69	522210	2.23
718	118	66	291525	1.78
719	100	51	190125	1.96
720	100	51	190125	1.96
721	100	51	190125	1.96
722	145	56	494325	2.57
723	100	51	190125	1.96
724	100	51	190125	1.96
mean	357	184	8000940	1.91
